# Skeletal Muscle Pathological Fat Infiltration (Myosteatosis) Is Associated with Higher Mortality in Patients with Cirrhosis

**DOI:** 10.3390/cells11081345

**Published:** 2022-04-14

**Authors:** Maryam Ebadi, Cynthia Tsien, Rahima A. Bhanji, Abha R. Dunichand-Hoedl, Elora Rider, Maryam Motamedrad, Vera C. Mazurak, Vickie Baracos, Aldo J. Montano-Loza

**Affiliations:** 1 Division of Gastroenterology and Liver Unit, University of Alberta, Edmonton, AB T6G 2X8, Canada; ebadi@ualberta.ca (M.E.); rbhanji@ualberta.ca (R.A.B.); rider@ualberta.ca (E.R.); 2 Ajmera Transplant Program, Department of Medicine, University of Toronto, Toronto, ON M5S 1A8, Canada; cynthia.tsien@gmail.com; 3 Division of Human Nutrition, University of Alberta, Edmonton, AB T6G 2P5, Canada; abha@ualberta.ca (A.R.D.-H.); motamedr@ualberta.ca (M.M.); vmazurak@ualberta.ca (V.C.M.); 4 Department of Oncology, Cross Cancer Institute, Edmonton, AB T6G 1Z2, Canada; vickie.baracos@ualberta.ca

**Keywords:** muscle quality, radiation attenuation, survival, muscle radiodensity, computed tomography

## Abstract

Myosteatosis (pathological fat accumulation in muscle) is defined by lower mean skeletal muscle radiodensity in CT. We aimed to determine the optimal cut-offs for myosteatosis in a cohort of 855 patients with cirrhosis. CT images were used to determine the skeletal muscle radiodensity expressed as Hounsfield Unit (HU). Patients with muscle radiodensity values below the lowest tertile were considered to have myosteatosis. Competing-risk analysis was performed to determine associations between muscle radiodensity and pre-transplant mortality. Muscle radiodensity less than 33 and 28 HU in males and females, respectively, were used as cut-offs to identify myosteatosis. In the univariate analysis, cirrhosis etiology, MELD score, refractory ascites, variceal bleeding, hepatic encephalopathy, sarcopenia and myosteatosis were predictors of mortality. Myosteatosis association with mortality remained significant after adjusting for confounding factors (sHR 1.47, 95% CI 1.17–1.84, *p* = 0.001). Patients with concurrent presence of myosteatosis and sarcopenia constituted 17% of the patient population. The cumulative incidence of mortality was the highest in patients with concomitant sarcopenia and myosteatosis (sHR 2.22, 95% CI 1.64–3.00, *p* < 0.001). In conclusion, myosteatosis is common in patients with cirrhosis and is associated with increased mortality. The concomitant presence of myosteatosis and sarcopenia is associated with worse outcomes.

## 1. Introduction

Advances in body composition assessment using cross-sectional computed tomography (CT) imaging have revealed radiologically recognized abnormalities in skeletal muscle. Sarcopenia (low muscle mass) and myosteatosis (pathological fat accumulation in muscle) are predominant muscle abnormalities that are associated with poor prognostication in patients with cirrhosis [1]. While the independent association between sarcopenia and poor outcomes both pre-and post-liver transplantation (LT) is well-documented [2], data on the clinical implications of myosteatosis as well as the concomitant presence of myosteatosis and sarcopenia in cirrhosis are limited.

Skeletal muscle radiodensity can be quantitatively assessed by CT in Hounsfield Units (HU) [3]. Pre-established CT radiodensity for skeletal muscle identification is in the range of −29 to 150 HU [4]. Lower skeletal muscle mean radiodensity has been linked to aberrant fat accumulation in the muscle [5]. Changes in muscle composition by lipid deposition can alter muscle quality, which is defined as the ratio of muscle strength to mass [6].

Emerging evidence suggests the prognostic significance of myosteatosis in predicting mortality [7,8] and hepatic encephalopathy [9,10] in patients with cirrhosis. BMI-dependent cut-off values established in cancer patients have been widely used to define myosteatosis in cirrhosis. Reduced muscle radiodensity has been defined as muscle radiodensity of <33 HU in patients with a BMI ≥ 25 kg/m^2^ and <41 HU in those with a BMI < 25 kg/m^2^ in cancer patients [11]. By applying these cancer-specific cut-offs for myosteatosis, 52% of patients with cirrhosis had myosteatosis [7]. The relevance of these cut-offs to patients with cirrhosis is debatable, as BMI measurements may be inaccurate in patients with fluid accumulation. Although psoas muscle radiodensity below 43.14 HU has been associated with higher 12-month mortality [12], it has limited prognostic precision in comparison to the whole muscle [13,14]. Another limitation is the lack of sex-specific cut-offs for myosteatosis, as the lipid storage capacity of skeletal muscle is higher in females compared to males [15].

Thus, we aimed to establish sex-specific cut-off values to delineate normal and low muscle radiodensity using one of the largest North American cohorts of patients with cirrhosis. Our secondary aims were to determine whether there was an association between myosteatosis and pre-LT mortality, independent of sarcopenia, in patients with cirrhosis and to explore the impact of the concurrent presence of myosteatosis and sarcopenia on pre-LT mortality.

## 2. Materials and Methods

### 2.1. Study Population

The Institutional Review Board of the University of Alberta has reviewed and approved this retrospective study (Pro00066572). Of 1104 adult patients with cirrhosis who had a CT image acquired as part of LT evaluation between January 2000 and August 2021, 249 patients were subsequently excluded. The exclusion criteria included the absence of cirrhosis, multiple organ transplantation or re-transplantation. A total of 855 patients with cirrhosis were included in this study.

### 2.2. Computed Tomography Image Analysis

The presence of sarcopenia and myosteatosis was assessed using the secondary analysis of CT images retrieved from the patients’ LT evaluation records. Abdominal CT scans taken at the 3rd. lumbar vertebra (L3) were assessed to estimate skeletal muscle cross-sectional area and radiodensity. Muscles including rectus abdominus, external/internal oblique muscles, transversus abdominus, psoas and paraspinal (quadratus lumborum, erector spinae) were quantified. A strong correlation has been reported between skeletal muscle areas taken at L3 with the whole-body muscle mass (*r*^2^ = 0.855, *p* < 0.01), making L3 a consistent landmark [16]. Standard HU thresholds of −29 to 150 HU [4] were applied to measure the cross-sectional area and mean radiodensity of skeletal muscle using Slice-O-Matic software (V4.2; Tomovision, Montreal, QC, Canada). The sum of skeletal muscle cross-sectional areas (cm^2^) was normalized to patient height in meters (m^2^) and reported as skeletal muscle index (SMI; cm^2^/m^2^). Sarcopenia was defined as SMI < 39 cm^2^/m^2^ in females and <50 cm^2^/m^2^ in males [17]. Skeletal muscle radiodensity was estimated as the mean value for the entire muscle cross-sectional areas.

### 2.3. Statistical Analysis

Descriptive statistics are reported as mean and standard deviation (SD) for continuous and frequency for categorical variables. Comparison between groups was performed using an independent *t*-test and the Pearson χ^2^ test. The correlation between SMI and muscle radiodensity was estimated using Pearson’s correlation coefficient (*r*) analysis.

The main outcome of this study was mortality, described as death prior to LT or delisting due to clinical deterioration. Mortality predictors were determined using the Fine–Gray sub-distribution hazard model rather than the conventional survival analysis. Competing risk analysis was used as it is a more robust approach and considers the presence of competing events (death and LT) [18]. The results of the univariate and multivariate Fine–Gray sub-distribution hazard analysis were reported as sub-distribution hazard ratios (sHRs) with 95% confidence intervals (CIs). The multivariable analysis included factors with a *p* < 0.10 in the univariate analysis. Parameter estimates of the Fine and Gray model were used to perform the cumulative incidence functions for mortality.

Muscle radiodensity was introduced into the model either as a continuous or nominal variable. Given the absence of linearity based on the optimal reference categories, we elected not to use a ROC-based criterion to reduce the risk of overestimation and optimism bias. Dichotomized variables are a better fit for clinical use and were made based on tertiles. In this exploratory study, patients with muscle radiodensity values below the lowest sex-specific tertile were considered as having myosteatosis. Stata 15.0 (StataCorp LLC) and SPSS 26.0 (SPSS for Windows, version 26.0, SPSS, Chicago, IL, USA) were used to perform the statistical analyses, and a *p*-value less than 0.05 was considered a statistically significant difference.

## 3. Results

### 3.1. Baseline Patients Characteristics

Sixty-three percent of the patients were male with a mean age of 56 ± 8 years and a MELD score of 15 ± 8. The etiology of cirrhosis was hepatitis C (39%), alcohol (25%), NASH (20%), autoimmune liver diseases (8%), hepatitis B (6%) and 42% of patients had concomitant hepatocellular carcinoma. Over the mean follow-up time of 24 ± 35 months, 369 patients died, 349 received LT and 142 patients were censored at the time of the last follow-up.

### 3.2. Myosteatosis and Mortality

Using competitive risk analysis, a more robust approach than Cox survival analysis, skeletal muscle radiodensity (sHR 0.98, 95% CI 0.96–0.99, *p* < 0.001) was a predictor of mortality in the univariate analysis (Table 1). Applying the lowest tertile as the reference group for myosteatosis, muscle radiodensity less than 33 HU in males and below 28 HU in females were statistically significant values that provide satisfactory discrimination of mortality risk between patients. Using these cut-offs, myosteatosis was identified in 34% of the patients. Myosteatosis was associated with the highest mortality risk after adjusting for factors, including autoimmune liver disease cirrhosis, MELD score, refractory ascites, variceal bleeding, hepatic encephalopathy and sarcopenia (sHR 1.47, 95% CI 1.17–1.84, *p* = 0.001; Table 1). The association between myosteatosis and mortality was independent of alcohol use disorder being the cause of cirrhosis.

**Figure 1:**
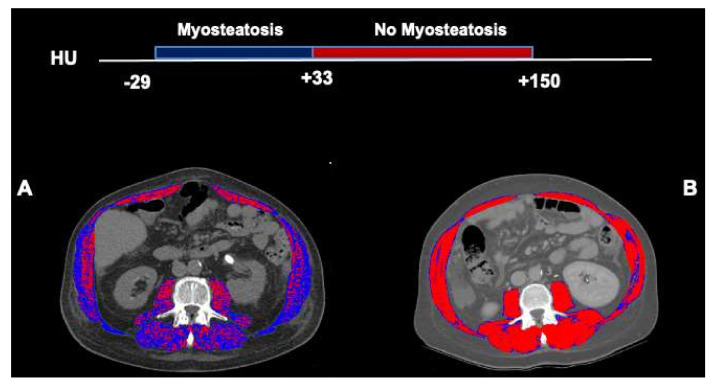
Abdominal computed tomography images taken at the 3rd. lumbar vertebra to quantify muscle radiodensity in patients with cirrhosis. Comparison of two male patients with cirrhosis and similar skeletal muscle index (58 cm^2^/m^2^). Skeletal muscle areas with high radiodensity (33 to 150) are shown in red, and low radiodensity muscle (−29 to 32) is shown in dark blue. In a patient with low mean muscle radiodensity (24 HU) or myosteatosis (**A**), the majority of the muscle areas are composed of the low attenuation muscle whereas, in a patient with normal muscle radiodensity (51 HU, no-myosteatosis), areas with the normal attenuation range are predominant (**B**).

In the imaging, myosteatosis is evidenced by an expansion of low radiodensity muscle areas. Figure 1 highlights the muscle radiodensity estimation at L3 from two male patients with cirrhosis and similar skeletal muscle index (58 cm^2^/m^2^). Figure 1A represents a patient with myosteatosis who had low muscle attenuation (24 HU), whereas Figure 1B shows a patient with a normal muscle radiodensity or no-myosteatosis (51 HU). Areas composed of low-radiodensity muscle (<33 HU) are predominant in Figure 1A, whereas, in Figure 1B, areas of normal-radiodensity muscle (≥33 HU) are prevalent.

### 3.3. Characteristics of Patients with Myosteatosis

A significant linear (*p* < 0.001), but weak (*r* = 0.37) correlation was observed between muscle radiodensity and SMI. Clinical features associated with myosteatosis are presented in Table 2. Patients with myosteatosis were more likely to have alcohol-related cirrhosis, were older, had higher MELD and BMI and had lower serum sodium. The frequency of refractory ascites and hepatic encephalopathy was also higher in these patients. Sarcopenia, defined as skeletal muscle index < 50 cm^2^/m^2^ in males and <39 cm^2^/m^2^ in females [17], was more frequent in patients with myosteatosis compared to their counterparts (50% vs. 32%, *p* < 0.001). No difference in the presence of diabetes or albumin levels was noticed between the two groups.

### 3.4. Skeletal Muscle Abnormality Phenotypes

**Figure 2:**
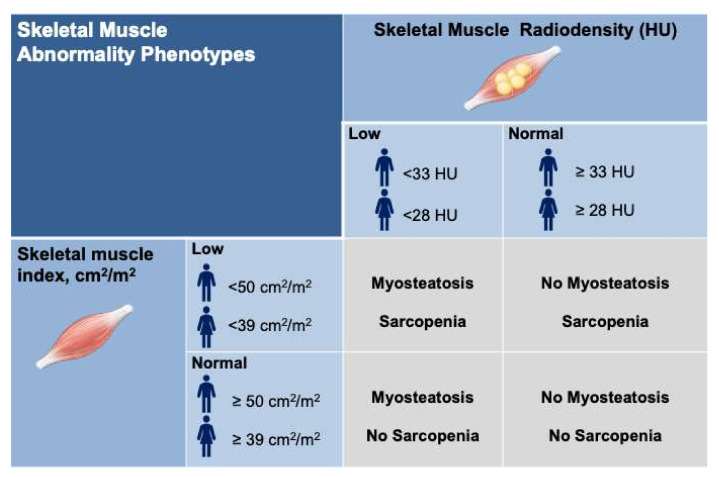
Classification of skeletal muscle abnormality phenotypes. Four-muscle abnormality phenotypes included those with no skeletal muscle abnormalities (45%), myosteatosis alone (17%), sarcopenia alone (21%) or concurrent sarcopenia and myosteatosis (17%). * Sarcopenia was defined using established cut-offs in patients with cirrhosis as skeletal muscle index < 50 cm^2^/m^2^ in males and <39 cm^2^/m^2^ in females [17]. Myosteatosis was established as muscle radiodensity <33 HU in males and <28 HU in females.

We divided the patients into four-muscle abnormality phenotypes based on various interactions between sarcopenia and myosteatosis (Figure 2); those with no skeletal muscle abnormalities (45%), myosteatosis alone (17%), sarcopenia alone (21%) or concomitant sarcopenia and myosteatosis (17%).

Patients with myosteatosis and without the presence of sarcopenia had a higher risk of mortality (sHR 1.62, 95% CI 1.21–2.16, *p* = 0.001; Table 3) relative to the patients with normal muscle. In multivariable competing risk analysis, the concomitant presence of myosteatosis and sarcopenia was associated with the highest risk of pre-LT mortality (sHR 2.22, 95% CI 1.64–3.00, *p* < 0.001; Table 3) when compared to patients with normal muscle and patients with either myosteatosis or sarcopenia alone.

**Figure 3:**
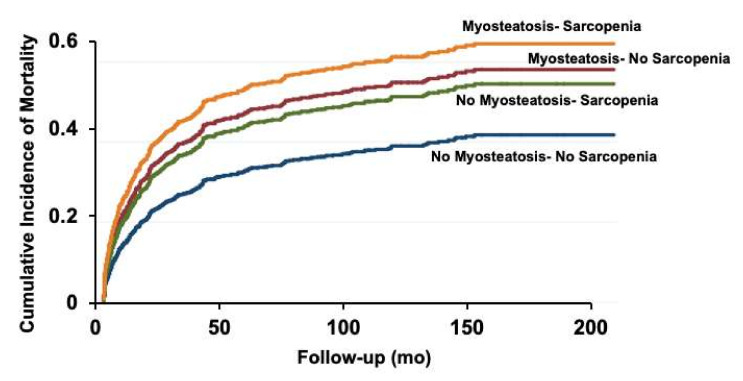
Cumulative incidence (Fine and Gray) of pre-liver transplant mortality in patients with various myosteatosis and sarcopenia interactions.

The cumulative incidence of pre-LT mortality was plotted for four-muscle abnormality phenotypes. Not surprisingly, the cumulative incidence of mortality was the highest in patients with concomitant sarcopenia and myosteatosis (Figure 3).

The cumulative incidence of pre-LT mortality was plotted for four-muscle abnormality phenotypes and compared using the sub-distribution hazard function. Patients with concomitant sarcopenia and myosteatosis had the highest cumulative incidence of pre-liver transplant mortality.

## 4. Discussion

The clinical implication of low muscle radiodensity (myosteatosis) in predicting mortality was investigated in one of the largest North American cohorts of 855 patients with cirrhosis. Patients with cirrhosis and low muscle radiodensity had a higher frequency of complications when compared to those with normal muscle radiodensity. Similar findings have been reported for the association between myosteatosis and risk of decompensation as well as survival in patients with cirrhosis in previous studies using cut-offs derived from cancer populations [7,9]. BMI was not a significant predictor of mortality in this cohort, demonstrating the importance of applying CT-derived muscle measurements. Our study provides cut-offs specific to patients with cirrhosis, which can be used to predict mortality risk pre-LT. CT scans are carried out consistently as part of LT evaluation, and secondary analysis of these available CTs requires no additional radiation exposure or cost.

Skeletal muscle radiodensity was associated with mortality in the univariate competitive risk analysis (HR 0.98, 95% CI 0.96–0.99, *p* < 0.001). This means that for every one HU increase in muscle radiodensity, there is a two percent decrease in the mortality risk. However, when patients were divided into tertiles, only the lowest tertile was significantly associated with mortality, suggesting that lower tertile captures patients with severe myosteatosis that is associated with higher mortality risk.

Patients with concomitant presence of myosteatosis and sarcopenia were found to have the worst survival after adjusting for clinically significant covariates. Both myosteatosis and sarcopenia were independent predictors of mortality, and the presence of both entities has additive effects as it deliberates the phenotype with the worst survival risk in cirrhosis. Application of sex-specific cut-offs developed in this study, only 17% of patients exhibited an overlapping presence of sarcopenia and myosteatosis, suggesting that these two conditions do not commonly co-exist in cirrhosis.

Myosteatosis signifies an alteration in the muscle quality due to the increased proportion of lipid accumulation in the muscle. Histological and biochemical analysis of the muscle collectively reveals an elevated level of triglyceride in muscle with low radiodensity, in the form of intermuscular adipose tissue, intramuscular adipose tissue and/or intramyocellular lipids [19,20,21]. Although myosteatosis in cirrhosis has been mainly defined by a low mean muscle radiodensity on CT, intermuscular adipose tissue cross-sectional areas or normalized radiodensity have also been previously applied as measures of myosteatosis [8,22]. It has been speculated that CT-measured IMAT may be less precise than mean muscle radiodensity as it constitutes only a small portion of the CT image and may be subject to inter-observer bias [8]. Of note, the composition of lipid, rather than the total amount of lipid accumulation per se might be important in the pathogenesis of myosteatosis [5].

Mechanisms of pathological lipid storage within the muscle in cirrhosis have not been identified but may be linked to metabolic aberrations associated with hepatic dysfunction. Hyperammonemia may have a significant role in the pathophysiology of myosteatosis in cirrhosis. Elevated skeletal muscle ammonia uptake can promote skeletal muscle mitochondrial dysfunction via diminished lipid oxidation [23], which results in the accumulation of lipid mediators and insulin resistance in muscle [24]. Other factors including diminished lipid storage capacity of subcutaneous adipose tissue [20,25] and age-related differentiation of muscle stem cells into adipocytes [26] have been suggested as potential mechanisms contributing to myosteatosis in various conditions. A better understanding of the mechanisms underlying myosteatosis is required to develop a treatment for the reversal of this skeletal muscle abnormality. The efficacy of ammonia-lowering treatments in reversing myosteatosis in cirrhosis requires further investigation.

In this study, mortality predictors were determined using competitive risk analysis rather than the conventional survival analysis, with LT as the competitive event. Risk overestimation is the main limitation of conventional survival analysis in the presence of competing events [18]. Limitations of this study include the extended period for the inclusion of the cases and the development of the radiological techniques over this period. Due to the retrospective nature of this study, we may have not addressed unknown confounders, and we were unable to evaluate metabolic characteristics. Though cut-offs established in this study need to be validated in larger prospective multi-center studies, to our knowledge, this study is one of the largest studies in North America demonstrating a relationship between myosteatosis and adverse outcomes in patients with cirrhosis.

## 5. Conclusions

In conclusion, low muscle radiodensity (low radiodensity on CT; myosteatosis) is associated with a worse prognosis, including mortality, and a higher frequency of complications in patients with cirrhosis. Though myosteatosis and sarcopenia present poor prognosis in cirrhosis, they are not considered in conventional prediction scores, such as the MELD or Child–Pugh scores. Including skeletal muscle abnormalities within the MELD score may improve prognostication in patients with cirrhosis. Timely recognition of reduced muscle mass or radiodensity by CT signifies targetable predictive factors. Further studies are needed to assess management, the impact of management on the maintenance of muscle quantity and quality and on long-term clinical outcomes.

## Figures and Tables

**Table 1:** Factors associated with mortality in univariate and multivariate competing risk analyses.

	Univariate		Multivariate	
Characteristics	sHR (95% CI)	*p*-Value	sHR (95% CI)	*p*-Value
Age (years)	0.99 (0.98–1.003)	0.15		
Sex, male	0.996 (0.81–1.23)	0.98		
Cirrhosis etiology				
- Alcohol	1.13 (0.90–1.43)	0.29		
- Hepatitis C	0.91 (0.74–1.12)	0.38		
- Hepatitis B	1.00 (0.64–1.56)	0.99		
- NASH	1.20 (0.95–1.53)	0.13		
- Autoimmune liver disease	0.62 (0.40–0.95)	0.03		
Albumin (g/L)	0.99 (0.97–1.01)	0.28	0.55 (0.36–0.85)	0.007
MELD score	1.03 (1.01–1.04)	<0.001	1.04 (1.03–1.06)	<0.001
Refractory Ascites	1.45 (1.13–1.88)	0.004	1.54 (1.15–2.08)	0.004
Sodium (mmol/L)	0.997 (0.98–1.01)	0.71		
Encephalopathy	1.65 (1.31–2.08)	<0.001	1.80 (1.37–2.36)	<0.001
Variceal bleeding	1.91 (1.38–2.64)	<0.001	1.57 (1.09–2.25)	0.02
Diabetes	0.85 (0.61–1.17)	0.31		
HCC	1.12 (0.91–1.37)	0.29		
BMI, kg/m^2^	1.01 (0.99–1.03)	0.20		
Skeletal muscle radiodensity, HU	0.98 (0.96–0.99)	<0.001		
Myosteatosis	1.56 (1.26–1.92)	<0.001	1.47 (1.17–1.84)	0.001
* Sarcopenia	1.43 (1.16–1.76)	0.001	1.55 (1.24–1.94)	<0.001

Abbreviations: BMI, body mass index; HU, Hounsfield units; HCC, hepatocellular carcinoma; MELD, model for end-stage liver disease; NASH, non-alcoholic steatohepatitis; sHR, sub-distribution hazard ratio. * Sarcopenia was defined using established cut-offs in patients with cirrhosis as skeletal muscle index < 50 cm^2^/m^2^ in males and <39 cm^2^/m^2^ in females [17]. Myosteatosis was established as muscle radiodensity <33 HU in males and <28 HU in females.

**Table 2:** Clinical features associated with myosteatosis.

Characteristics	Myosteatosis (n = 295)	No Myosteatosis (n = 560)	*p*-Value
Age (years)	57 ± 8	56 ± 9	0.02
Sex, male	189 (64)	346 (62)	0.55
Cirrhosis etiology			
- Alcohol	95 (32)	120 (21)	0.001
- Hepatitis C	103 (35)	233 (42)	0.07
- Hepatitis B	7 (2)	48 (9)	<0.001
- NASH	66 (22)	105 (19)	0.21
- Autoimmune liver disease	21 (7)	50 (9)	0.43
Albumin (g/L)	32 ± 7	32 ± 6	0.37
MELD score	17 ± 9	14 ± 7	<0.001
Refractory Ascites	100 (34)	133 (24)	0.002
Sodium (mmol/L)	135 ± 8	136 ± 5	0.006
Encephalopathy	135 (46)	183 (33)	<0.001
Variceal bleeding	47 (16)	108 (19)	0.26
Diabetes	46 (16)	65 (12)	0.11
HCC	112 (38)	243 (43)	0.14
BMI, kg/m^2^	28 ± 6	27 ± 5	<0.001
Skeletal muscle radiodensity, HU	25 ± 6	39 ± 6	<0.001
* Sarcopenia	148 (50)	177 (32)	<0.001

Abbreviations: BMI, body mass index; HCC, hepatocellular carcinoma; HU, Hounsfield units; MELD, model for end-stage liver disease; NASH, non-alcoholic steatohepatitis; * Sarcopenia was defined using established cut-offs in patients with cirrhosis as skeletal muscle index < 50 cm^2^/m^2^ in males and <39 cm^2^/m^2^ in females [17]. Myosteatosis was established as muscle radiodensity <33 HU in males and <28 HU in females.

**Table 3:** Association between 4-muscle abnormality phenotypes and mortality.

	Univariate		Multivariate	
Characteristics	sHR (95% CI)	*p*-value	sHR (95% CI)	*p*-Value
Age (years)	0.99 (0.98–1.003)	0.15		
Sex, male	0.996 (0.81–1.23)	0.98		
Cirrhosis etiology				
- Alcohol	1.13 (0.90–1.43)	0.29		
- Hepatitis C	0.91 (0.74–1.12)	0.38		
- Hepatitis B	1.00 (0.64–1.56)	0.99		
- NASH	1.20 (0.95–1.53)	0.13		
- Autoimmune liver disease	0.62 (0.40–0.95)	0.03	0.55 (0.35–0.84)	0.006
Albumin (g/L)	0.99 (0.97–1.01)	0.28		
MELD score	1.03 (1.01–1.04)	<0.001	1.04 (1.03–1.06)	<0.001
Refractory Ascites	1.45 (1.13–1.88)	0.004	1.53 (1.14–2.07)	0.005
Sodium (mmol/L)	0.997 (0.98–1.01)	0.71		
Encephalopathy	1.65 (1.31–2.08)	<0.001	1.79 (1.36–2.34)	<0.001
Variceal bleeding	1.91 (1.38–2.64)	<0.001	1.58 (1.10–2.29)	0.01
Diabetes	0.85 (0.61–1.17)	0.31		
HCC	1.12 (0.91–1.37)	0.29		
BMI, kg/m^2^	1.01 (0.99–1.03)	0.20		
4-Muscle abnormalities phenotype				
(1) No myosteatosis-No sarcopenia	Ref			
(2) Myosteatosis-No sarcopenia	1.61 (1.22–2.12)	0.001	1.62 (1.21–2.16)	0.001
(3) Sarcopenia-No Myosteatosis	1.46 (1.10–1.92)	0.008	1.71 (1.28–2.28)	<0.001
(4) Myosteatosis-Sarcopenia	1.92 (1.44–2.55)	<0.001	2.22 (1.64–3.00)	<0.001

Abbreviations: BMI, body mass index; HU, Hounsfield units; HCC, hepatocellular carcinoma; MELD, model for end-stage liver disease; NASH, non-alcoholic steatohepatitis; sHR, sub-distribution hazard ratio. * Sarcopenia was defined using established cut-offs in patients with cirrhosis as skeletal muscle index < 50 cm^2^/m^2^ in males and <39 cm^2^/m^2^ in females [17]. Myosteatosis was established as muscle radiodensity <33 HU in males and <28 HU in females.

## Data Availability

The datasets generated and analyzed during the current study are not publicly available but are available from the corresponding author on reasonable request.
